# Highlights on the Management of Oligometastatic Disease

**DOI:** 10.4103/JIPO.JIPO_24_19

**Published:** 2020-02-05

**Authors:** Salem M. Alshehri, Khaled Alkattan, Ahmed Abdelwarith, Hussain Alhussain, Shaker Shaker, Majed Alghamdi, Hossam Alassaf, Ali Albargawi, Manal Al Naimi, Ameen Alomair, Saif Althaqfi, Adnan Alhebshi, Majid Alothman, AbdulRahman Jazieh

**Affiliations:** 1 Department of Oncology, King Abdulaziz Medical City, Ministry of National Guard Health Affairs, Riyadh, Saudi Arabia; 2 King Saud bin Abdulaziz University for Health Sciences, Ministry of National Guard Health Affairs, Riyadh, Saudi Arabia; 3 King Faisal Specialist Hospital and Research Center, Riyadh, Saudi Arabia; 4 Department of Medicine, King Saud University Hospital, Riyadh, Saudi Arabia; 5 Department of Radiation Oncology, King Fahad Medical City, Riyadh, Saudi Arabia; 6 Al Baha University, Al Baha, Saudi Arabia; 7 Department of Radiation Oncology, King Fahad Specialist Hospital, Dammam, Saudi Arabia; 8 Radiation Oncology Unit, John Hopkins Aramco Healthcare, Dharan, Saudi Arabia

**Keywords:** Oligometastases, stereotactic ablative body radiotherapy, stereotactic body radiotherapy

## Abstract

**Purpose:** The understanding of oligometastatic disease (OMD) is rapidly evolving and with this comes the ability to utilize a number of modalities that excel in the localized control of disease. It has been identified that there are no clear guidelines based on high-level evidence to standardized approaches toward the management of OMD. These highlights have been developed to provide a road map for all health-care professionals who are involved in the management of OMD to support standardized patient care. **Methods:** The Saudi Lung Cancer Guidelines Committee is a part of the Saudi Lung Cancer Association which, in turn, is part of the Saudi Thoracic Society. Considering that lung cancer constitutes a major proportion of OMD prevalence, the committee took the initiative to develop national highlights to support the management of OMD within Saudi Arabia. The committee members are national clinical leaders who collaborated with international expertise to establish these highlights to serve as a general clinical pathway in the management of OMD. **Results:** Standardization of the indications to diagnose oligometastases and patient selection criteria including ineligibility criteria for treatment are the basis of the highlights. Treatment approaches including surgical and the variety of radiotherapeutical options are discussed in relation to specific oligometastatic sites. Acceptable measurements for response to treatment and the future for the treatment of OMD conclude the development of the highlights. **Conclusion:** These are the first national highlights addressing this important disease in oncology. The implementation of these highlights as guidelines requires a robust multidisciplinary team and access to specific technology and expertise. These highlights are based on the most recent findings within the literature but will require repeated review and updating due to this rapidly evolving field in disease management.

## Introduction

Overall, metastatic malignancies are associated with a poor prognosis, where systemic chemotherapy, immunotherapy, and molecular targeted therapy are the standards of treatment. Oligometastatic status has an increasing significance in the selection in appropriate treatment strategies available for metastatic disease. Immunotherapy and radiation therapy namely stereotactic ablative radiotherapy (SABR) have proven in literature to have a synergistic effect on cancer cells.[1–3]

The biologic basis for the clinical discrepancy between widespread and oligometastatic disease (OMD) may include different primary tumor microenvironments, fitness of the migrant cancer cells, and the hospitability of host sites.[4] Tumor biology likely differs for oligometastatic versus widely metastatic disease, with variation in genetic signatures and expression profiles.[5,6]

The concept of OMD was introduced by Hellman and Weichselbaum in 1995 to describe a state in which the extent of metastases is limited in number and location, for which a curative therapeutic strategy may be indicated.[7] Oligometastases are typically defined as a limited number of metastases that are not rapidly evolving and can be contained by aggressive management. The number of limited metastatic sites discussed within the literature is either one to three metastatic sites or five and fewer metastases.[8] Knowing that up 40%–55% of non-small cell lung cancer (NSCLC) patients present with distant metastases, 70% of them had up to two metastatic lesions and 50% had three or fewer metastatic sites.[9] In a single retrospective study, 26% of patients with metastatic lung cancer had five or fewer metastatic lesions.[10] Common primary tumor sites for subsequent development of OMD include NSCLC, colorectal cancer, breast, prostate, soft-tissue sarcoma, and renal cell carcinoma. Common sites of extracranial oligometastases include lung, liver, bone, adrenals, and lymph nodes.

The defined OMD categories are:[11]

- Synchronous: Initially presented at diagnosis
- Metachronous or oligo-recurrence: Recurring metastatic site(s) after initial primary treatment
- Oligoprogressive: Disease progression at few metastatic sites, while response or stable disease (SD) at other sites.

The challenge in the management of OMD is distant recurrence and if local recurrence occurs, it will mostly take a place within the first 2 years. Identifying OMD depends on the reliability of advanced diagnostic imaging to accurately point out the number of metastatic sites. Advanced imaging such as positron emission tomography-computed tomography (PET/CT) and magnetic resonance imaging (MRI) have enhanced the ability to assess patients for metastatic disease.

Local consolidative therapy for selected oligometastatic NSCLC has shown to prolong overall survival (OS) and progression-free survival (PFS) in comparison to maintenance therapy or observation.[12,13] Aggressive local treatment for oligometastases includes brain, pulmonary, and hepatic metastasectomy; stereotactic radiosurgery (SRS); stereotactic body radiotherapy (SBRT), also known as SABR; radiofrequency ablation; and cryoablation, either alone or combined with systemic chemotherapy. The multiple treatment modalities available have been heavily researched over a significant period of time. It is important to consider that the response and outcome of OMD to local treatment modalities is influenced by the biology of the primary tumor type.

SBRT is defined as the administration of highly conformal and image-guided external beam radiotherapy in an accurate and precise way, delivered in limited number of fractions (< 5).[14] With the advance in technology, SBRT has become feasible and safe to apply large “ablative” doses of radiation therapy in very few treatments, which makes this practical technique a widely adopted. This subject is an active area for ongoing worldwide clinical trials which may change the current practice of treating oligometastases.

In patients with metastatic NSCLC, retrospective and prospective studies have demonstrated improved outcomes for patients who received treatment to all known metastases.[15–19]

OMD treatment in prostate cancer has evolved; the Oriole trial demonstrated prolonged PFS in the SABR arm in comparison to the observation arm.[20]

Population-based analysis has demonstrated modest improvements in OS in metastatic breast cancer when treating patients with combination chemotherapy, reporting outcomes in over 1500 women at M. D. Anderson Cancer Center.[17] Extended PFS was demonstrated in small number of patients with 3.1% and 1.6% at 5 and 15 years, respectively.[17] This prolonged PFS of this subset of patients who received standard systemic treatment suggests that interventions to cure or control OMD may further increase this small proportion of long-term survivors, supporting local consolidation therapy approaches in this setting. In 2015, in an international survey among more than 1000 radiation oncologists, 61% reported using SBRT to treat oligometastases.[21]

Recently, the (SABR-COMET) trial has shown that SABR treatment is associated with an improvement of OS in OMD.[22]

### Highlight 1: Oligometastases: Indications and patient selection for localized therapy

Since the 1960s, multiple expert groups proposed indications for the surgical resection of metastatic tumors. The original criteria proposed by Thomford et al*.* in 1965 are still utilized in clinical practice in an expanded format.[23]

Currently, the criteria include:[23]

- Controlled primary malignancy
- No evidence of extrathoracic progressive metastases
- All tumors are resectable, with adequate remaining pulmonary reserve
- No available alternative local treatment modality with a lower morbidity risk.

#### Pearls in oligometastatic disease

- Metastatic lesion(s) or site(s) of disease count individually
- Only active lesion(s) or site(s) of disease count
- Metastatic lesion(s) is a synonym to metastatic site(s); both terms are reported in literature[12,13,19,24]
- Number of organs involved cannot be solely a determinant factor.[25]

#### Conditions for patient ineligibility

Any patient who has one of the following conditions is NOT an ideal candidate for curative SBRT or aggressive local treatment, and an alternative treatment modality is recommended for management (e.g., systemic treatment or palliative treatment):[26,27]

- Primary tumor progression (breast, prostate, or lung)
- Indistinct borders of metastatic lesion(s)
- Initial palliative radiotherapy to same metastatic site. Whole-brain radiotherapy (WBRT) is an exception
- Complete response (CR) to systemic treatment (i.e. no existing target)
- Malignant pleural effusion
- Spinal cord compression (clinically or radiologically)
- Femoral bone metastases
- Previously irradiated targets within 3 cm[28]
- Pregnant or lactating women
- Severe, active comorbidity, for example, unstable angina and/or congestive heart failure requiring hospitalization within the last 6 months; acute bacterial or fungal infection requiring intravenous antibiotics; respiratory illness requiring hospitalization; and severe hepatic disease, defined as a diagnosis of Child–Pugh Class B or C hepatic disease
- HIV-positive patients with CD4 count < 200 cells/μL
- End-stage kidney disease.

### Highlight 2: Treatment approaches

#### Stereotactic body radiotherapy recommendations

It is highly recommended that the decision-making process for SBRT as an aggressive local treatment modality follows a dedicated workflow, which requires the following:[26,27]

- Evaluation by a multidisciplinary tumor board including but not limited to a radiation oncologist, medical oncologist, thoracic surgeon, neurosurgeon, hepatobiliary surgeon, pathologist, and radiologist
- Detailed history and physical examination
- Pathology confirmation of malignancy
- A precise assessment of Zubrod/Eastern Cooperative Oncology Group (ECOG) Performance Status ≤ 2 within planned treatment time, i.e. Karnofsky Performance Status (KPS) of > 60%
- Age ≥ 18 years old
- Controlled primary tumor.
- Imaging workup to document oligometastatic status of the disease:
  - CT scans of chest, abdomen, and pelvis; CT/MRI brain; as well as whole-body PET/CT to confirm oligometastatic status
  - High-definition local imaging by MRI to be utilized for image fusion and target delineation purposes.
- Informed consent is required prior to treatment
- Negative serum/urine pregnancy test within 14 days for females of childbearing age.
- Laboratory workup to be obtained, to assure adequate bone marrow, renal, and liver function/reserves including:
  - Complete blood count (CBC)/differential defined as follows: (not absolute contraindication)
  - Absolute neutrophil count ≥ 500 cells/mm^3^
  - Platelets ≥ 50,000/mm^3^
  - Hemoglobin ≥ 8.0 g/dl
  - Liver enzymes are < 3X (upper normal limits) for liver metastases
  - Liver functions must be documented
  - Adequate renal functions.
- HIV-positive patients are candidates for SBRT provided they are under treatment with highly active antiretroviral therapy and have a CD4 count ≥ 200 cells/μl.

#### Surgical recommendations: Resection of limited metastases

There is surgical data demonstrating long-term disease control and survival in patients treated with metastasectomy from sarcoma and breast cancer among other primary tumors. Patients presenting with spinal cord compression from solid tumors who undergo surgical decompression in addition to radiation have improved ambulatory function, continence, and survival compared to radiation monotherapy.[29–31]

Fong et al. published their experience with metastasectomy of hepatic oligometastases on 456 patients with colorectal cancer treated between 1985 and 1991.[32] The treatment was well tolerated with low mortality and a postresection median survival of 46 months and 38% with a 5-year survival. A later publication showed that 22% of these patients achieved 10-year survival and were effectively cured of their disease.[33] Subsequent studies led to hepatic resection for oligometastases from colorectal cancer becoming the standard of care in the absence of a prospective clinical trial in an era prior to oxaliplatin and irinotecan chemotherapy backbones.[34] This provides a preliminary evidence base to suggest that a subset of patients with limited metastatic disease may be curable with localized treatment beyond chemotherapy.

In the pre-SBRT era, the treatment with a curative intent for metastatic tumors in the lung was pulmonary metastasectomy. Pulmonary metastasectomy would be appropriate for only 15%–25% of patients with pulmonary metastases.[35] A systematic review and meta-analysis showed that OS ranged from 27% to 68% after pulmonary metastasectomy for metastatic colorectal cancers.[36] Risk factors for poor OS included short disease-free interval, multiple lesions, and elevated prethoracotomy carcinoembryonic antigen.[37]

Recommendations for preoperative evaluation:

- Detailed history and physical examination
- Assessment of respiratory symptoms
- Appropriate imaging modality to assess extrathoracic metastases
- High-quality imaging to assess number and location of pulmonary metastases and assist in surgical planning
- Thorough functional assessment including questioning of the patient and family about the capabilities of completing activities of daily living
- Pulmonary function testing is a crucial component to the preoperative evaluation of those who are undergoing an anatomic resection of metastatic lesions.

The optimum history taking starts with an assessment of respiratory symptoms, although up to 90% of patients with pulmonary metastases will be asymptomatic due to the nonobstructing peripheral nature of their disease.[38] Postoperative diffusion capacity (diffusion capacity for carbon monoxide) and forced expiratory volume at 1 second must be determined, as they are important predictors of operative risk, postoperative complications, and mortality. Sublobar resection (either wedge resection or segmentectomy) is most often used for patients undergoing metastasectomy; consideration must be given to the potential cumulative parenchymal loss in the setting of multiple lesions. Patients who have been subjected to three or more surgical resections are at risk of pulmonary functional losses similar to those undergoing a lobectomy.[35] Each of the Expanded Thomford criteria must be met before offering surgery. The Thomford criteria do not take into account the prognosis, whereas the concept of oligometastases includes the prognosis of possible cure.

In research studies, pulmonary metastasectomy has been primarily evaluated by OS; there are additional prognostic factors to consider: tumor doubling time, disease-free interval, and number and distribution of pulmonary metastases.[39] OS represents the entire duration of various treatments, including local ablation, and does not depend on cure but rather on the length of time that the patients are alive. For patients with multiple metastases, there is no consensus to define how many lesions are too many. Achieving complete resection with adequate pulmonary reserve is vital; therefore, adequate pulmonary reserve mandates the evaluation of the number of nodules, consideration of the locations, and estimation of the postoperative pulmonary function.

Five-year survival rates are variable depending on the number of metastatic lung lesions with a single metastatic focus (43% 5-year survival), while it is 34% with those with two to three metastases, it is 27% for patients with three or more metastases.[39] The role of mediastinoscopy is still arguable, hence the impact of nodal disease was most likely related to the histology of the primary malignancy.[39,40] Currently, if a lesion can be completely cleared while allowing for adequate remaining function, then resection can be pursued even if the lesions are numerous, bilateral, or if anatomic resection such as segmentectomy or lobectomy is required. In the case of potential pneumonectomy, a thorough discussion of alternative therapies, in a multidisciplinary setting, is mandated prior to embarking upon surgery. The indication for pulmonary metastasectomy to prolong OS remains an unresolved issue that necessarily requires a randomized prospective study, but in the era of quick-paced drug development, it is considered almost impossible to identify the significance of the local therapy in any analysis of OS.

In conclusion, the criteria prior to proceed for pulmonary metastasectomy would be:

- Confirmed number of metastatic lesions by imaging CT scans and PET-CT
- Adequate pulmonary function test
- Good Performance Status (PS) < 2
- Decision to be undertaken by multidisciplinary team
- Consider combining several local therapy modalities, for example, surgery and SBRT
- Treatment-naïve patients with mutation-driven disease should consider initial systemic treatment.

### Highlight 3: Classification of oligometastases by site

#### Intracranial oligometastases

Patients are eligible for aggressive local therapy if they meet all of the following criteria:

- Favorable prognosis using preferably grade point average score, if not then Recursive Partitioning Analysis: Classes I and II[41,42]
- Good PS using ECOG ≤ 2 or KPS >70%
- Stable, controlled, primary disease (recent restaging is required)
- Lymphoma and germ cell tumors are not eligible for SRS.

Criteria for the number and size of metastases are described below:

##### Solitary brain metastasis

There is strong evidence within the literature supporting local treatment for limited metastatic disease in the context of intracranial metastases. Randomized trials have demonstrated improvements in disease control and OS for patients treated with surgical resection or SRS in addition to WBRT.[43,44]

Surgical resection is also indicated for large and/or symptomatic brain metastasis as it provides immediate relief compared to radiotherapy unless surgery cannot be performed due to patient- or disease-related issues.

Postoperative management would be viewed according to the following points:

- Following surgery for solitary brain metastases, local stereotactic radiotherapy (SRT) (1–5 fractions) to tumor bed is the standard of care[45]
- For patients with solitary brain metastasis who cannot undergo or refuse surgery, SRS using a single fraction is indicated for tumor sizes up to 4 cm and fractionated SRT (up to 5 fractions) is preferred for tumors >4 cm or tumors located at eloquent areas in the brain[46]
- WBRT for intact or resected solitary brain metastasis has fallen out of favor given the negative impact on cognitive functions without improvement of OS[47]
- Doses and target volumes for single-fraction SRS can be tailored as per the Radiation Therapy Oncology Group 9508.[44]

WBRT is preserved for the following circumstances:

- Presence of spread pattern leptomeningeal disease
- Presence of multiple metastatic lesions (number is variable from center to center)
- Specific pathology (hematologic malignancy, germ cell tumors, small cell lung cancer [SCLC], and lymphoma).

##### Multiple brain metastases

Management of multiple brain metastases would be viewed according to the following points:

Workup:

- Patients with intracranial metastases ideally simulated with stereotactic frame
- It is recommended that all patients with intracranial metastases have primary MRI planning with thin cuts (1–2 mm) which will be co-registered with the planning of thin-cut CT (not > 2 mm).

Treatment:

- Surgery is indicated for resectable symptomatic brain metastasis even in the presence of multiple other metastases which can be treated with SRS as a primary treatment or SRS as postoperatively
- SRS (single fraction) is indicated for brain metastases up to four lesions in number and up to 4 cm in size
- Treating more than four lesions with SRS is not supported by strong evidence; however, it can still be used by an expert radiation oncologist based on clinical judgment[48]
- For brain metastatic lesions > 4 cm or for lesions located at eloquent areas in the brain, fractionated SRT is preferred[49]
- In case of > 4 brain metastatic lesions, WBRT can be used; however, SRT can still be also used by an expert radiation oncologist using clinical judgment for up to ten brain metastatic lesions[48]
- Doses for fractionated SRT range from 20 to 35 Gy in five daily and consecutive fractions. Target volumes are at radiation oncologist's discretion
- For resected brain metastases, volumes for fractionated SRT may be performed according to the consensus guidelines[50]
- If the metastases are removed surgically, SRS or SRT to the resection cavity plus 1–2 mm margins may improve local control[51]
- Radiation dose varies according to the volume and location of the metastasis where 13–24 Gy in a single fraction can be given.

Hypofractionation is recommended in selected cases with large lesions or large resection cavity or where the constraints to the surrounding critical structures cannot be achieved with a dose of single fraction. The hypofractionated dose varies between 21 and 30 Gy in 3–5 fractions. The volume of whole brain receiving 12 Gy is preferred to be < 20%–30%.

##### Spinal oligometastases

Patients are eligible for aggressive local therapy if they meet all of the following criteria:

- Good PS using ECOG ≤ 2 or Karnofsky Performance Status (KPS) > 70%
- Stable, controlled primary disease (recent restaging required)
- SCLC, lymphoma, and germ cell tumors are excluded
- Limited number of involved vertebral levels.

#### Indications for surgery

The indications for curative surgical intervention in the context of spinal oligometastases are summarized in the following circumstances:

- Presence of spinal instability signs as per the Spinal Instability Neoplastic Score (SINS) criteria, score >6[52]
- Significant vertebral compression fracture (> 50%)
- Presence of epidural disease deforming thecal sac and contacting the spinal cord (grade 1c), as per the Bilsky grading system[53]
- Malignant symptomatic spinal cord compression, especially single level, as it is supported by Level I evidence when compared to palliative external beam radiotherapy alone[31]
- Surgical treatment is crucial for decompression and stabilization[54]
- Alternatively, minimally invasive vertebroplasty or kyphoplasty can be considered for patients with significant vertebral compression fracture (> 50%) if surgical decompression and stabilization procedure is deemed not suitable[55]
- Following surgery, if patient is not a good candidate for SBRT, conventional external beam radiotherapy is indicated (20–30 Gy in 5–10 fractions).

#### Eligibility criteria for spinal stereotactic body radiotherapy

The following criteria have to be present in any patient before considering eligible for spinal SBRT:

- Involvement of ≤ 3 adjacent vertebrae or multiple nonadjacent levels (separated by at least one vertebral level)
- Absence or low-grade epidural disease
- Absence of spinal instability signs as per the SINS criteria[52,56,57]
- No significant compression fracture (> 50%) as it can increase the risk of further fracture. Vertebroplasty or kyphoplasty can be performed before considering these patients for SBRT[56]
- SBRT can be used in the postoperative setting for selected patients.[58]

#### Ineligibility criteria for spinal stereotactic body radiotherapy

- Myeloma or lymphoma
- Nonambulatory patients
- Compression fracture (50% loss of vertebral body height)
- Spinal cord compression or displacement
- Epidural compression within 3 mm of the spinal cord
- Rapid neurologic deterioration
- Bony retropulsion
- Recent radiation to the same spinal area
- MRI is medically contraindicated
- Patients allergic to contrast dye used in MRIs or CT scans.

### Spinal stereotactic body radiotherapy specifications and requirements

Before implementing a spinal SBRT program in an institution, certain criteria (including staff, technique, and logistics) have to be assured and confidently available:

- It is highly advised that SBRT to be performed by an expert radiation oncologist using image-guided radiotherapy (IGRT) technique. The required technique is described by Foote et al. (2011)[57,59,60]
- Spinal SBRT is indicated in all pathologies except SCLC, lymphoma, and germ cell tumors
- Spinal metastases referred to any spinal/paraspinal site(s) evolve within vertebral bodies or 1 cm of the vertebral bodies
- Spinal SBRT can target metastases on:
  - Vertebral body only
  - Vertebral body and pedicle only
  - Posterior elements “arc” only
- The target volumes may be chosen at the discretion of the treating radiation oncologist based on the extent of tumor involvement and the available techniques
- Gap of 3 mm or more between the edge of the epidural metastasis and edge of the spinal cord is recommended in the presence of epidural extension
- Osseous metastases planning guidelines are used for metastases arising in the ribs within 1 cm of the edge of the vertebral body
- Paraspinal mass ≤ 5 cm that is contiguous with spine metastasis
- Pretreatment and planning thin slice T1- and T2-weighted MRIs are required to assess the extent of disease and position of the cord and for treatment planning
- Proper fusion of MRI and CT simulation is required
- Myelogram can be used to improve cord visualization, especially in the presence of metal instrumentation that can obscure the cord[60]
- Paraspinal or rib disease can be treated with SBRT if it is within 5 cm from spinal cord. Caution is advised to protect organs at risk (OAR) such as lungs and kidneys
- The target volumes may be chosen as per the consensus guidelines for intact spinal metastases SBRT and postoperative SBRT[59,60,61]
- Priority is always set to protect the cord while achieving the highest percentage of target volume coverage without overdosing the cord. Underdosing part of target volume is always required in the case of epidural disease
- Preferred SBRT dose is 24 Gy in two consecutive fractions or 16 Gy in a single fraction[61,62]
- Spinal cord dose limit is 12 Gy point maximum in single fraction and 17 Gy in two fractions.[61,62]

#### Intrathoracic oligometastases

Some patients with intrathoracic oligometastatic are suitable for aggressive local management. Anatomically, intrathoracic metastases are defined as lesions within the anatomic space below the thoracic inlet at the level of the top of the sternal notch and above the diaphragm.

Eligibility criteria:

- Age >18 years
- Good PS ECOG ≤2 or (KPS >60)
- Controlled or stable primary disease (recent restaging is required preferably by PET/CT)
- All pathologies are accepted except SCLC, lymphoma, and germ cell tumors
- Thoracic locations include:[26]
  - Rib metastases adjacent to mediastinal or cervical structures
  - Scapular metastases within the thorax adjacent to lung parenchyma
  - Sternal bone metastases.

Number of lesions:

- 1–5 metastases for both lungs
- 1–3 metastases for single lung
- 1–3 mediastinal
- Diameter of 1–5 cm.
- Dose fractionation changes according to metastatic site(s) location
- Respiratory motion management including abdominal compression, active breathing control, breath hold, end expiratory gating, or fiducial marker tracking is highly recommended
- Localization Using Daily IGRT as an SBRT protocol, for example, technologies such as cone-beam CT (CBCT)
- For central lesions defined as lesions within 2 cm from proximal bronchial tree (trachea and major bronchus), the dose would be 50 Gy in five fractions
- For peripheral lesions, the dose prescribed in large body of literature is 48 Gy in four fractions
- Attention should be paid to critical organ sites namely normal lung preserve, spinal cord, great vessels, esophagus, brachial plexus, and proximal bronchial tree
- Constraints for four-fraction regimen: Spinal cord volume receiving 20.8 Gy to be < 0.35 cc and maximum point dose not to exceed 20.8 Gy. The volume of proximal bronchial tree receiving 15.6 Gy not to exceed 4cc and a maximum point < 34.8 Gy[26,63]
- Constrains for five-fraction regimen: spinal cord volume receiving 28, 22, and 15.6 Gy not to exceed 0.03, 0.35, and 1.2 cc, respectively. The volume of proximal bronchial tree receiving 40 Gy not to exceed 0.03 cc[64,65]
- The total volume of both lungs receiving 11.6 Gy and 12.4 Gy < 1500 cc and 1000 cc, respectively, when using the four-fraction regimens[26]
- The total volume of both lungs receiving 12.5 Gy and 13.5 Gy < 1500 cc and 1000 cc, respectively, when using the four-fraction regimens[66]
- In case of two or more lung lesions, the lung volume receiving 20 Gy < 15%.[63]

#### Head-and-neck oligometastases

Patient selection for head-and-neck SBRT must be discussed and evaluated by conducting a multidisciplinary tumor board meeting, which includes at least a radiation oncologist, a medical oncologist, an ear, nose and throat surgical oncologist, a pathologist, and a radiologist.

SBRT is increasingly used to treat a variety of head-and-neck tumors, primary or metastatic, as a result of its highly conformal dose distribution and stereotactic spatial accuracy in delivery. Most of the data regarding the use of SBRT in head-and-neck treatment are for primary and recurrent tumors due to limited data for metastatic disease to the head-and–neck region. Extrapolating from the success of SBRT in treating primary/recurrent head-and-neck cancer is a safe and wise method to be used in treating metastases to the head-and–neck region using SBRT.[67,68] The potential use of SBRT is mainly at surgically inaccessible areas and where negative margins are difficult to achieve without causing significant functional morbidity.

For any patient to be suitable for aggressive local management of oligometastases, the following points have to be considered:

- Detailed history/physical examination, which includes the PS, smoking or alcohol use, nutritional status, oral hygiene, and human papillomavirus status
- Imaging workup to document metastasis and evaluate the primary disease and CT scans of head and neck, chest, abdomen, and pelvis with whole-body PET/CT and local MRI
- Controlled primary disease
- Attention has to be paid to the dose/fractionation based on target tumor volume and dose to the normal surrounding structures
- Based on the published literature, tumors < 25 cc can be treated with SBRT using doses up to 40–45 Gy over five fractions provided that critical organs can be spared and there is no large blood vessel involvement. For larger tumors, a more protracted course of 40–45 Gy over 10–15 fractions is advised.[69] These SBRT regimens are typically delivered every other day[68]
- Caution on dosing is advised. The risk of carotid blowout in the re-irradiation setting ranges from 3% to 20%. The carotid blowout range is 1%–20%.[69]

#### Hepatic oligometastases

Patient selection for liver SBRT must be discussed and evaluated in multidisciplinary tumor board meeting which includes at least a radiation oncologist, a medical oncologist, a hepatobiliary surgeon, a pathologist, a gastroenterologist, an interventional radiologist, and a radiologist. The patient has to be suitable for aggressive local management of oligometastases. Historically, surgical hepatic metastasectomy has a proven track record with 5-year survival rates of 50%–60% for selected patients. As most patients with liver metastases remain ineligible for surgery, alternative local treatment modalities, such as SBRT, radiofrequency ablation, microwave ablation, or radiolabeled microspheres, have shown some benefit.

A patient is a candidate for aggressive local management for oligometastases if he/she meets the following majority criteria:[27,28]

- Good PS ECOG ≤ 2
- Controlled or stable primary disease (recent restaging required)
- Child–Pugh class A/low B
- All pathologies except SCLC, lymphoma, and germ cell tumors
- Lesion(s) assessed on a contrast-enhanced liver CT, MRI, or PET/CT within 6 weeks
- Five or less liver lesions
- Metastasis size ≤ 8 cm
- Adequate bone marrow function, based on CBC/differential obtained within 2 weeks
- The normal liver is defined as that portion of liver not radiographically involved by gross tumor
- Assessment of anatomical and physiological reserve to estimate the function of “residual liver.”

In terms of constraints:[27,28,63]

- All patients required to preserve at least 700 cc of normal liver
- No more than 30% of the normal liver to receive more than 27 Gy
- No more than 50% of normal liver to receive over 24 Gy
- At least 700 cc of normal liver not to receive more than 15 Gy.

#### Abdomino-pelvic oligometastases

Anatomically, abdomino-pelvic oligometastases are defined as lesions within the anatomic space below diaphragm superiorly and the genitourinary diaphragm inferiorly including the peritoneal and retroperitoneal spaces, but not including liver, osseous, or spinal metastases.[28] Those patients have to be suitable for aggressive local management of oligometastases. Attention is advised for specific critical organs such as the small bowel and duodenum. Patients need to meet all of the following criteria:

- Age > 18 years
- Good PS ECOG ≤2 or (KPS > 60)
- Controlled or stable primary disease (recent restaging required preferably PET/CT)
- Good high-resolution local imaging to clearly identify the target
- All pathologies except SCLC, lymphoma, and germ cell tumors.

#### Adrenal gland oligometastases

Adrenal gland metastases are present in up to 50% of patients with lung cancer; isolated adrenal gland metastases with NSCLC are rare, 1%–6%.[70,71] Adrenalectomy can be considered in carefully selected patients who have unilateral, isolated adrenal metastases and an excellent PS. SBRT is a noninvasive approach for adrenal gland metastases. The total dose range is 25–48 Gy delivered within a total number of five fractions.[72,73] Precise and accurate delivery using several IGRT technologies is highly recommended, for example, MRI fusion, fiducial insertion for cyber knife tracking, and four-dimensional CT. Higher local control rate (> 70%) can be achieved with total biologically equivalent dose (BED) > 60 Gy and (> 90%) if total BED ≥90 Gy.

#### Bone (osseous) oligometastases

Osseous oligometastases are defined as lesions within any osseous structure including part of the axial skeleton, but not vertebral locations.

The following specific locations should be considered for better location definitions according to the NRG BR001 trial:[28]

- Rib metastases within 1 cm of the vertebral bodies are classified as spinal metastasis
- Rib/scapular metastases within the thorax adjacent to lung parenchyma are classified as lung metastasis location
- Rib/osseous metastases adjacent to mediastinal or cervical structures are classified as the mediastinal/cervical lymph node location
- Rib metastases adjacent to the liver are classified as the liver location
- Rib metastases adjacent to the stomach/abdominal wall are classified as the intra-abdominal location
- Sternal bone metastasis is considered as mediastinal/cervical lymph node location.

#### Oligometastases management plan

##### Workflow prior to treatment

- Multidisciplinary team service compiling all services involved in OMD management
- It is recommended that all services provide all available modalities of local therapy to be involved
- Dedicated recognized team and clinic to implement and monitor the outcome
- Affiliation to internationally recognized body is highly recommended.

To implement SABR service in an institution, multiple settings have to be assured:[27]

- Dedicated SABR quality assurance meeting for SBRT treatment
- SABR plans must meet target dose levels with respect to OAR constrains
- Planned SABR dose must be verified by both the physicist and the treating physician
- Strict adherence to dose constraints is highly recommended
- IGRT as CBCT should be used to verify patient positioning prior to each treatment
- Direct tumor localization for soft-tissue tumors is recommended, if not feasible, then reliable soft-tissue surrogates are recommended
- Repeated CBCT is recommended if treatment delivery time exceeds 25 min
- A final CBCT is optional after completion of treatment
- Head-and-neck oligometastasis simulation is usually based on contouring the gross tumor volume (GTV) using thin CT cuts of 1–1.5 mm, preferably with MRI fusion. The clinical target volume (CTV) margins vary depending on the intent and the location of disease. For oligorecurrence salvage for cure in the skull base where there is a high stability, a CTV of 3 mm would be adequate, while at least 5 mm is required in other areas. For palliation, it is acceptable to have no margins beyond the GTV to minimize toxicity. The PTV varies from one center to another depending on image guidance technology and setup variability, ranging from 1 to 5 mm expansion.

### Highlight 4: Response assessment: Present and future

#### Outcome evaluation

The clinical outcome is evaluated by comparing the disease response utilizing pretreatment and posttreatment imaging (CT, 18fluorodeoxyglucose [FDG]-PET-CT, or MRI). Imaging frequency posttreatment would be performed every 3 months by CT initially. Occasionally, ^18^FDG-PET-CT is needed after treatment for better discrimination between necrosis and avid viable lesion.

The radiological response is based on the RECIST Criteria:[74]

- CR: Disappearance of the lesions at CT scan
- Partial remission: Reduction of > 30%
- SD: Any response < 30% to nearly unchanged disease
- Progression of disease: Any growing lesion not clearly ascribable to fibrosis.

The incidence of toxicity can be graded according to the most recent National Cancer Institute Common Terminology Criteria for Adverse Events scale.[75]

### Ongoing trials

The use of SABR for oligometastases has increased with overall control rate achieving 80%, which would be reflected on the OS along with multimodality cancer treatment.[76] Internationally, a number of studies recently addressed the role, safety, and potential benefits of local therapy in OMD including SABR-COMET (NCT01446744), STOMP (NCT01558427), and NRG-BR001 (NCT02206334).[22,28,77] Several clinical trials addressing the role of SABR for oligometastases are ongoing and accrual is still a challenge. The SABR-COMET 10 is a Phase 3 clinical trial addressing the outcome of SABR in terms of survival and quality of life for selected patients with OMD.[78] NRG-BR002 is a randomized trial comparing SBRT and/or surgery of all metastases versus standard of care for patients with oligometastatic breast cancer (NCT02364557).[79]

### Future directions: Stereotactic body radiotherapy and the immune response

SBRT is the potential trigger to enhance tumor-specific immunity, and thus “prime” the immune system to immunotherapy. In addition to the well-known effect of SBRT in terms of DNA damage and direct cell death, SBRT appears to stimulate CD8+ T-cell responses. Optimal SBRT dose and timing of therapies is still the subject of ongoing research. Several ongoing clinical trials combining SBRT with immunotherapy will be hopefully an area of promising future in oncology world.[80] Several studies have suggested that SBRT or hypofractionated regimens is superior to conventional fractionation for the activation of antitumor CD8+ T-cell response (1–3). Synergistic antitumor effect has been observed in preclinical models when RT is combined with immunotherapy. With immunotherapy revolution being as a standard treatment for many solid tumors, there is growing interest in combining immunotherapy and SBRT as a means to improve response rates.
